# LFCseq: a nonparametric approach for differential expression analysis of RNA-seq data

**DOI:** 10.1186/1471-2164-15-S10-S7

**Published:** 2014-12-12

**Authors:** Bingqing Lin, Li-Feng Zhang, Xin Chen

**Affiliations:** 1 School of Biological Sciences, Nanyang Technological University, Singapore; 2 School of Physical and Mathematical Sciences, Nanyang Technological University, 637371 Singapore; 3 Institute of Statistical Science, Shenzhen University, 518060 Shenzhen China

**Keywords:** Differential expression, Nonparametric, RNA-seq

## Abstract

**Background:**

With the advances in high-throughput DNA sequencing technologies, RNA-seq has rapidly emerged as a powerful tool for the quantitative analysis of gene expression and transcript variant discovery. In comparative experiments, differential expression analysis is commonly performed on RNA-seq data to identify genes/features that are differentially expressed between biological conditions. Most existing statistical methods for differential expression analysis are parametric and assume either Poisson distribution or negative binomial distribution on gene read counts. However, violation of distributional assumptions or a poor estimation of parameters often leads to unreliable results.

**Results:**

In this paper, we introduce a new nonparametric approach called LFCseq that uses log fold changes as a differential expression test statistic. To test each gene for differential expression, LFCseq estimates a null probability distribution of count changes from a selected set of genes with similar expression strength. In contrast, the nonparametric NOISeq approach relies on a null distribution estimated from all genes within an experimental condition regardless of their expression levels.

**Conclusion:**

Through extensive simulation study and RNA-seq real data analysis, we demonstrate that the proposed approach could well rank the differentially expressed genes ahead of non-differentially expressed genes, thereby achieving a much improved overall performance for differential expression analysis.

## Background

RNA sequencing (RNA-seq), which applies high-throughput DNA sequencing technologies to directly sequence complementary DNAs (cDNAs), has completely transformed the way in which transcriptomes are studied. In particular, it permits the quantitative analysis of gene expression and transcript variant discovery, which was not made possible with the previous microarray technologies [1,2]. RNA-seq is increasingly being used to investigate a wide range of biological and medical questions, *e.g*., in genomics research [3,4] and in clinic use [5,6].

In RNA-seq experiments, millions of short fragments (reads) are sequenced from samples and aligned back to a reference genome. The expression level of a feature (gene, exon or transcript) is then measured by the read count which is the number of short reads that map to the feature. When RNA-seq measurements are made for multiple samples from different biological conditions, a question of particular interest is to identify genes/features that are differentially expressed across conditions. This is the primary aim of RNA-seq differential expression analysis.

There have been a number of statistical approaches proposed for differential expression analysis of RNA-seq data, and they broadly fall into two categories: parametric or nonparametric. In [7,8], the over-dispersed RNA-seq data is transformed so that the Poisson distribution can be used to model read counts. edgeR [9,10], DESeq [11], and sSeq [12] instead assume the negative binomial distribution on read counts--a flexible probability model allowing a larger variance than mean. The differences among these three approaches lie mainly in their different ways to estimate the dispersion parameter. EBSeq [13] and baySeq [14] also assume the negative binomial distribution, but they were cast within an empirical Bayesian framework. All the above parametric approaches are generally very efficient when the distributional assumption holds. However, violation of distributional assumptions or a poor estimation of parameters often leads to unreliable results. NOISeq [15] is a data-adaptive nonparametric approach that uses both log fold changes and absolute expression differences as test statistics. It is effective in controlling the false discovery rate (FDR) while being robust against the sequencing depth. SAMseq [16] is another nonparametric approach that utilizes a Wilcoxon statistic. It estimates the false discovery rate by a permutation plug-in procedure and thus is not sensitive to outliers in the data. Recently, an efficient algorithm based on a Markov random field model, called MRFSeq, was developed [17]. Different from previous methods, MRFSeq takes advantage of the additional gene co-expression data to effectively alleviate the selection bias of differentially expressed genes against genes with low read counts. For more discussions and comparisons of these differential expression analysis methods, we refer readers to [18] and [19].

In this paper, we propose a new data-driven nonparametric approach called LFCseq for differential expression analysis of RNA-seq data. Basically, it is based on a similar principle to NOISeq, but uses only log fold changes as the test statistic. To conduct a statistical test for each gene, LFCseq estimates a null or noise probability distribution by contrasting log fold changes for a selected set of genes at similar expression levels. In contrast, NOISeq relies on a null distribution estimated from all genes within an experimental condition regardless of their expression levels. However, as we shall demonstrate later, the null distribution of log fold changes varies considerably for genes at different expression levels, which makes the results from NOISeq less reliable.

## Methods

### Notation

Although biological experimental designs may vary greatly, RNA-seq data generated for differential expression analysis can all be written into a matrix **N**, whose element *N_ij _*is the number of reads mapped to gene *i *in sample *j *from an experimental condition *A *or *B*. Without ambiguity, we also use *A *(and *B*, respectively) to denote the set of samples *j *under the condition *A *(and *B*, respectively). That is, if j∈A, it indicates that sample *j *is under the experimental condition *A *rather than condition *B*. Let *x_i _*be a binary random variable indicating whether gene *i *is differentially expressed between two conditions *A *and *B*. We have *x_i _*= 1 if gene *i *is differentially expressed (DE) and *x_i _*= 0 if gene *i *is not differentially expressed (non-DE).

Typically, only a few samples are available in current RNA-seq experimental data; however, there could instead have up to tens of thousands of genes under examination. In the present study, we limit our discussions to two experimental conditions only, although our proposed approach can be extended to three or more conditions.

### Normalization

Since different samples may have different sequencing depths, the read counts *N_ij _*are not directly comparable across samples before being properly normalized [20,21]. A simple normalization scheme is to divide the read counts by the sample library size and gene length [20]. However, this total-count normalization was shown to be problematic, as the normalized read count of a gene is adversely affected by expression levels of all the other genes [11,21,22].

Many sophisticated normalization procedures have been proposed, including the trimmed means of M values (TMM) normalization in edgeR [22], quantile normalization [21], a 'median' normalization method in DESeq [11] and a goodness-of-fit method in PoissonSeq [7]. In our experiments below, we use the goodness-of-fit method to normalize read counts. It defines the sequencing depth for sample *j *as ${\hat{d}}_{j}=\sum_{i\in S}N_{ij}\text{/}\sum_{i\in S}\sum_{j}N_{ij}$, where *S *is a half set of genes that are least differentially expressed between two conditions as estimated by a Poisson goodness-of-fit statistic [7]. The normalized read count *n_ij _*is subsequently computed as $n_{ij}=N_{ij}\text{/}{\hat{d}}_{j}$.

### LFCseq

Let ${\bar{n}}_{i}^{A}$ and ${\bar{n}}_{i}^{B}$ be the means of the normalized read counts for gene *i *of samples under conditions *A *and *B*, respectively. That is, ${\bar{n}}_{i}^{A}=\frac{1}{\left|A\right|}{\text{Σ}}_{j\in A}n_{ij}$ and ${\bar{n}}_{i}^{B}=\frac{1}{\left|B\right|}{\text{Σ}}_{j\in B}n_{ij}$. In LFCseq, we use the log fold change of mean read counts, i.e.,

$$L_{i}={\text{log}}_{2}\frac{{\bar{n}}_{i}^{A}}{{\bar{n}}_{i}^{B}},$$

as the statistic to test differential expression. Because there are usually only a small number of samples under one condition, no read counts could be reliably identified as outliers. Therefore, we choose to use the mean instead of the median of read counts in the above definition (as NOISeq did). However, when there is obvious evidence that the outliers of read count exist or when the number of samples is large enough, median may be a better choice than mean. On the other hand, to avoid the division by zero, genes with zero read counts in all samples are removed from the analysis, and the zero counts are replaced by 0.5 for the rest of genes, as in [15].

We try to build a null or noise distribution for log fold changes by contrasting gene read counts within the same condition. To this end, we first divide the samples within a same condition into two groups of almost equal size. Let *A*_1 _and *A*_2 _be the two resulting groups of samples under condition *A *such that *A *= *A*_1 _∪ *A*_2 _and $|A_{1}|=\left\lceil \frac{\left|A\right|}{2}\right\rceil$. As we did in the preceding paragraph, let ${\bar{n}}_{i}^{A_{1}}=\frac{1}{\left|A_{1}\right|}\Sigma_{j\in A_{1}}n_{ij}$ and ${\bar{n}}_{i}^{A_{2}}=\frac{1}{\left|A_{2}\right|}\Sigma_{j\in A_{2}}n_{ij}$. They are the means of the normalized read counts for gene *i *within each group of samples. Then, the log fold change of read counts between two groups *A*_1 _and *A*_2 _is computed as

$$L_{i}^{A_{1}\cup A_{2}}={\text{log}}_{2}\frac{{\bar{n}}_{i}^{A_{1}}}{{\bar{n}}_{i}^{A_{2}}}.$$

When *|A| ≤ *7, we may compute the log fold change value $L_{i}^{A_{1}\cup A_{2}}$ for all the possible partitions of *A *into *A*_1 _and *A*_2_. However, when *|A| >*7, we compute it only for 20 random partitions in order to reduce the computational cost. Finally, we pool all these log fold change values together, and denote the resulting collection by $L_{i}^{A}$. By applying the same procedure as above, we can obtain a collection *LB *of log fold change values of read counts for gene *i *within condition *B*.

Given a gene *i*, we define its *neighborhood *as a set of genes with similar expression strength across conditions. Specifically, we define the neighborhood *N*(*i*) of gene *i *as $N\left(i\right)=\left\{i^{\prime }:|{\bar{n}}_{i^{\prime }}^{A\cup B}-{\bar{n}}_{i}^{A\cup B}|<\in_{i}\right\}\}$, where ${\bar{n}}_{i}^{A\cup B}=\frac{1}{\left|A\cup B\right|}\sum_{j\in A\cup B}n_{ij}$ and $\in_{i}$ set to a value such that *N *(*i*) would contain a predefined number of genes (default 50 genes). Then, we build a null fold change distribution *L_i _*for gene *i *by using

$$L_{i}=\underset{i^{\prime }\in N\left(i\right)}{⋃}L_{i^{\prime }}^{A}\cup L_{i^{\prime }}^{B}$$

Note that this null distribution is gene-specific, as it takes into account only genes from the neighborhood of gene *i*. A special case of the above proposed approach is obtained when the neighborhood of a gene includes all the genes in a sample under investigation.

With the log fold change *L_i _*of read counts of gene *i *between two conditions and a null fold change distribution *L_i_*, we approximate the probability of gene *i *being not differentially expressed as the fraction of points from *L_i _*that correspond to a larger absolute fold change value than *|L_i_|*. Therefore, we may write this probability as

$P\left(x_{i}=0|n_{ij},\forall j\right)=\frac{\left|\left\{l:|l|>|L_{i}|,l\in L_{i}\right\}\right|}{\left|L_{i}\right|}$.

**Figure 1:**
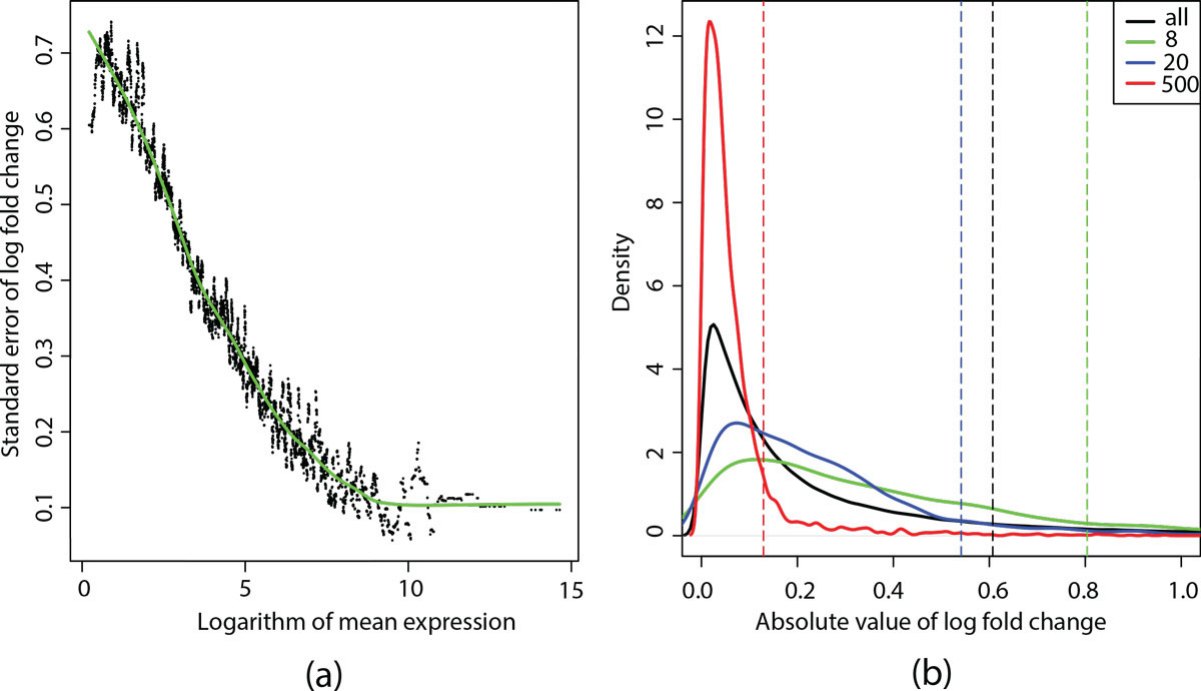
**Standard error and distribution of log fold changes within conditions on MAQC dataset**. (a) The scatter plots depict the standard errors of the null distribution *L_i_*. The neighborhood of each gene *i *contains 50 genes with similar expression strength. The green line is fitted by using R function "lowess". (b) There are three curves (green, blue, and red) representing the null distributions of log fold changes estimated from genes with the normalized read counts of around 8, 20, 500, respectively. The black curve represents the null distribution estimated from all genes regardless of their expression levels. Each dashed line indicates the 90% quantile of the respective distribution.

The above proposed approach to estimate the probability of a gene being non-DE is motivated by a previous observation in [11] that the squared coefficient of variation (i.e., the ratio of the variance to the mean squared) decreases as gene expression levels increase. We further found that the standard error of the null distribution *L_i _*decreases considerably (from 0.7 down to 0.1) as gene expression levels increase, as demonstrated in Figure 1(a). It clearly tells us that using a common null distribution to approximate the probability of genes being DE or non-DE, regardless of their expression levels, is not sufficient or appropriate. Therefore, we choose to group genes at similar expression levels and estimate the null fold change probability distribution based only on genes within the same group. As shown in Figure 1(b), the estimated null distributions vary substantially across different groups of genes. In general, the null distributions from genes of lower expression levels tend to shift to the right with heavier tails.

LFCseq is implemented in R and publicly available at http://www1.spms.ntu.edu.sg/~chenxin/LFCseq/.

### Relation to NOISeq

LFCseq was developed based on a similar principle to the nonparametric approach NOISeq [15]. It is worth pointing out their major differences. First, NOISeq uses not only the log fold change *L_i _*but also the absolute difference *|D_i_| *of mean read counts as the statistics to test gene *i *for differential expression, where the absolute difference *|D_i_| *is defined as $\left|D_{i}|=|{\bar{n}}_{i}^{A}-{\bar{n}}_{i}^{B}\right|$. Second, NOISeq estimates the null joint probability distribution (*L, D*) by computing the log fold change *R *and absolute difference *d *for every pair of samples within a same condition (in contrast to random partitions of samples within a condition into two subsets in LFCseq such as *A *= *A*_1 _∪ *A*_2_) and for every gene (in contrast to genes only in the neighborhood in LFCseq). Consequently, a common null distribution is applied to all genes in NOISeq to compute the probability of a gene being DE. That is,

$$P_{\text{NOISeq}}\left(x_{i}=1|n_{ij},\forall j\right)=\frac{\left|\left\{\left(l,d\right):|l|<|L_{i}|,|d|<|D_{i}|,\left(l,d\right)\in \left(L,D\right)\right\}\right|}{\left|\left(L,D\right)\right|}$$

## Results and discussion

### Datasets

We test the performance of LFCseq on two simulated and three real RNA-seq datasets, and compare it with six existing parametric and nonparametric approaches, including NOISeq, SAMseq, edgeR, DESeq, sSeq and EBSeq (see Additional file 1 for their running R codes).

**Simulation 1**. In this simulated dataset, there are a total of 20,000 genes and their read counts are generated from a negative binomial distribution under each condition *A *or *B*,

$$N_{ij}\sim NB\left(\mu_{ij},\sigma_{ij}^{2}\right)$$

where *µ_ij _*and $\sigma_{ij}^{2}$ are the mean and variance, respectively. As in [10], we further let *µ_ij _*= E{*N_ij _*} = *q_iA _d_j _*under condition *A *and *µ_ij _*= E{*N_ij _*} = *q_iB _d_j _*under condition *B*, where *q_iA _*and *q_iB _*represent the true expression values of gene *i *under condition *A *and *B*, respectively, and where *dj *represents the sequencing library size of sample *j*. For the variance, we let $\sigma_{ij}^{2}=\mu_{ij}+\phi_{i}.\mu_{ij}^{2}$, where *φ_i _*is the dispersion parameter of the negative binomial distribution. As a typical setting, 30% of the genes are simulated to be differentially expressed, among which 70% are set to be up-regulated. The library size factors are generated from the uniform distribution *d_j _~ U *(0.5, 1.5). We consider three different sample sizes *|A| *= *|B| *= 2, 5 and 8 under each condition.

**Simulation 2**. We generate read counts for 20,000 genes using the same procedure as above in Simulation 1, except that the parameter values of *q_iA_, q_iB _*, and *φ_i _*are randomly sampled with replacement from the experimental Bottomly's dataset [23]. Thus we expect this setting is more realistic than the previous one in Simulation 1.

**MAQC dataset**. MAQC dataset [24] contains two RNA sample types, Stratagene's human universal reference RNA (UHR) and Ambion's human brain reference RNA (brain). Each sample type has seven replicates. In this dataset, 844 genes have been assayed by the quantitative real-time polymerase chain reaction (qRT-PCR). As in [21], a gene is considered as differentially expressed if the log fold change ratio of its cycle threshold values exceeds 2 or as non-differentially expressed if this log fold change ratio is smaller than 0.2. As a result, we obtain 235 DE genes and 53 non-DE genes from the qRT-PCR gold-standard to assess the performance of the proposed approach.

**Griffith's dataset**. Gene expression is compared between two human colorectal cell lines [25], MIP101 and MIP/5-FU, of the fluorouracil (5-FU)-resistant and -nonresistant phenotype, respectively. qRT-PCR measurements were made for 94 genes. A two-tailed t-test was applied to identify DE and non-DE genes with a cutoff point 0.05, which left 83 DE genes and 11 non-DE genes for performance evaluation.

**Sultan's Dataset**. Gene expression of two human cell lines, Ramos B and HEK 293T, were compared using RNA-seq [26]. In this dataset, there are two replicates for each cell line. See Additional file 1 for further details of these testing datasets.

### Evaluation criteria

We evaluate the performance of LFCseq from the following two aspects. First, we evaluate its ability to discriminate between DE genes and non-DE genes by ranking genes in order of significance for differential expression between conditions. With the gene ranking list, we plot a receiver operating characteristic (ROC) curve and compute the area under the curve (AUC) to measure the overall discriminating ability. Then, LFCseq is compared with six other approaches in terms of AUC without imposing any arbitrary cutoffs. For LFCseq, we rank genes in increasing order of the probability *P *(*x_i _*= 0 *| n_ij _, ∀j*). For three parametric approaches that assumed the negative binomial distribution (edgeR, DESeq, sSeq), genes are ranked according to their estimated nominal p-values. For SAMseq, we use the false discovery rates (FDR) estimated by a permutation plug-in method and, for NOISeq and EBSeq, we use their estimated probabilities of genes being differentially expressed for ranking. Second, we evaluate the experimental results of LFCseq in terms of precision, sensitivity, and F-score. These evaluation metrics are defined as follows: PRE (precision) = TP/(TP+FP), SEN (sensitivity) =TP/(TP+FN), and FS (F-score) =2 *× *PRE *× *SEN*/*(PRE + SEN), where TP, FP, and FN are the number of true positives, the number of false positives and the number of false negatives, respectively. Note that the metric F-score is the harmonic mean of sensitivity and precision and thus measure the overall differential expression inference performance of a method. In general, the higher the F-score, the better the inference performance. In order to compute precision and sensitivity scores, all the approaches used their respective default settings to call a list of DE genes. Specifically, LFCseq, NOISeq, and EBSeq used a probability cutoff of 0.1, 0.8, and 0.95, respectively. SAMseq used a FDR cutoff of 0.05, while edgeR, DESeq, and sSeq all used a p-value cutoff of 0.05 after adjusted for multiple testing.

### Performance on simulated data

**Figure 2:**
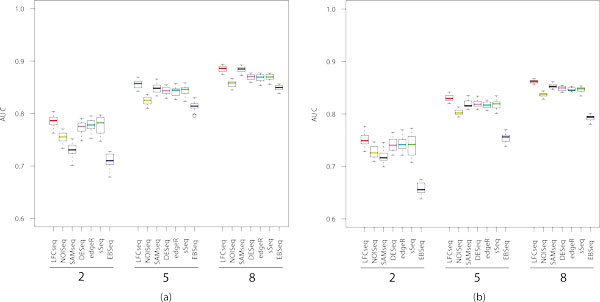
**Areas under the ROC curves for Simulation 1 and 2**. The boxplots summarize the AUCs averaged over 20 repetitions. Each panel corresponds to a different number of replicates, 2, 5 or 8. (a) Simulation 1. (b) Simulation 2.

Figure 2 shows the boxplots of AUC values for Simulation 1 and 2, averaged over twenty repetitions. We can clearly see that our proposed approach LFCseq achieved larger AUC values than any other tested method in both simulation settings, especially in the cases where the number of replicates is small. For example, in Simulation 1, LFCseq achieved the average AUC values of 0.785, 0.856, and 0.885 for the experiments with 2, 5 and 8 replicates, respectively. In comparison, the corresponding AUC values are 0.754, 0.825, and 0.856 from NOISeq, and 0.778, 0.842, and 0.868 from edgeR. Notably, EBSeq obtained the lowest AUC values in all tests, presumably due to its focus on the identification of DE isoforms instead of DE genes. While LFCseq and NOISeq are based on a similar principle to identify DE genes, we can see that LFCseq performed significantly better than NOISeq. This implies that the gene-wise null distributions of log fold changes (used in LFCseq) provide a more accurate model than a common null distribution for all genes (used in NOISeq). edgeR, DESeq, and sSeq are three parametric approaches assuming the negative binomial distribution. Although they applied different methods to estimate the dispersion parameter, their AUC values are actually very close to each other.

**Figure 3:**
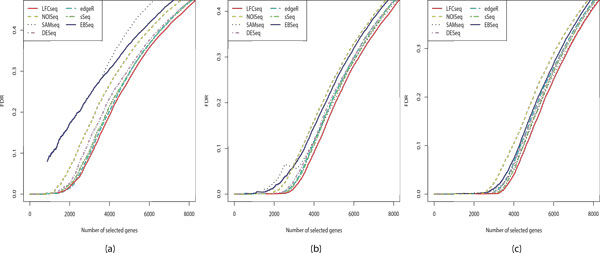
**False discovery rate curves for Simulation 1**. (a) 2 replicates per condition. (b) 5 replicates per condition. (c) 8 replicates per condition. The FDR curves for Simulation 2 can be found in Additional file 1.

Figure 3 plots the curves of false discovery rates (FDR) for the experiments in Simulation 1. As we can see, the FDR curve of LFCseq always stays below any other curve in all the tests. It indicates that when we fix a same number of DE genes to be called by each method, LFCseq will achieve the lowest false discovery rate (i.e., the lowest number of false positives). In other words, LFCseq has the improved ability to rank truly DE genes ahead of non-DE genes. SAMseq suffered significantly high false discovery rates in cases of two replicates under each condition. However, its rates get closer to those of LFCseq as the number of replicates increases.

The experimental results of precision, sensitivity and F-scores are summarized in Table 1 and in Table S1 in Additional file 1 for Simulation 1 and 2, respectively. Clearly, LFCseq has the best overall performance as it achieved the highest F-scores in all the tests. NOISeq achieved relatively higher precision scores than LFCseq. However, its sensitivity scores are much lower than those of LFCseq so that its overall performance becomes inferior to LFCseq. For example, in Simulation 1, the F-scores of LFCseq are 0.57, 0.72, and 0.78 for the tests with 2, 5, and 8 replicates, respectively. They are 0.11, 0.27, and 0.33 greater than the corresponding F-scores of NOISeq. SAMseq did not call any DE genes in the experiments with 2 replicates. This is not surprising considering that the power of the Wilcoxon test is generally low with a few replicates. However, it is not clear why sSeq did not call any DE genes either, while the other two similar parametric approaches edgeR and DESeq performed relatively well in both precision and sensitivity.

**Table 1:** Precision, sensitivity and F-score for Simulation 1.

Methods	PRE	SEN	FS	PRE	SEN	FS	PRE	SEN	FS
	|*A*|=|*B*|=2			|*A*|=|*B*|=5			|*A*|=|*B*|=8		
			
LFCseq	0.88	**0.42**	**0.57**	0.93	**0.59**	**0.72**	0.93	**0.68**	**0.78**
NOISeq	0.91	0.29	0.44	**1.00**	0.29	0.45	**1.00**	0.29	0.45
SAMseq	NA	0.00	NA	0.96	0.37	0.53	0.96	0.62	0.75
DESeq	**0.98**	0.20	0.34	0.99	0.47	0.63	0.98	0.58	0.73
Edger	0.96	0.32	0.48	0.94	0.55	0.70	0.93	0.63	0.75
sSeq	NA	0.01	NA	0.97	0.52	0.68	0.94	0.63	0.76
EBSeq	0.72	0.37	0.49	0.94	0.46	0.62	0.97	0.53	0.69

The numbers of replicates per condition are 2, 5 and 8, respectively. The highest precision, sensitivity and F-scores achieved are highlighted in bold. The corresponding results obtained in Simulation 2 can be found in Additional file 1.

In addition, we compared LFCseq with a simple hypergeometric test (SHGT) when the numbers of replicates per condition are 5 and 8 in Simulation 1. In the simple hypergeometric test, the null distribution for gene *i *is built on the randomly permuted samples of gene *i *between conditions A and B, instead of using the neighborhood *N *(*i*). From Figure S8 and Table S2 in Additional file 1, it can be seen that LFCseq performs better than SHGT in the terms of both AUC values and F-scores.

### 0.1 Performance on real data

**Figure 4:**
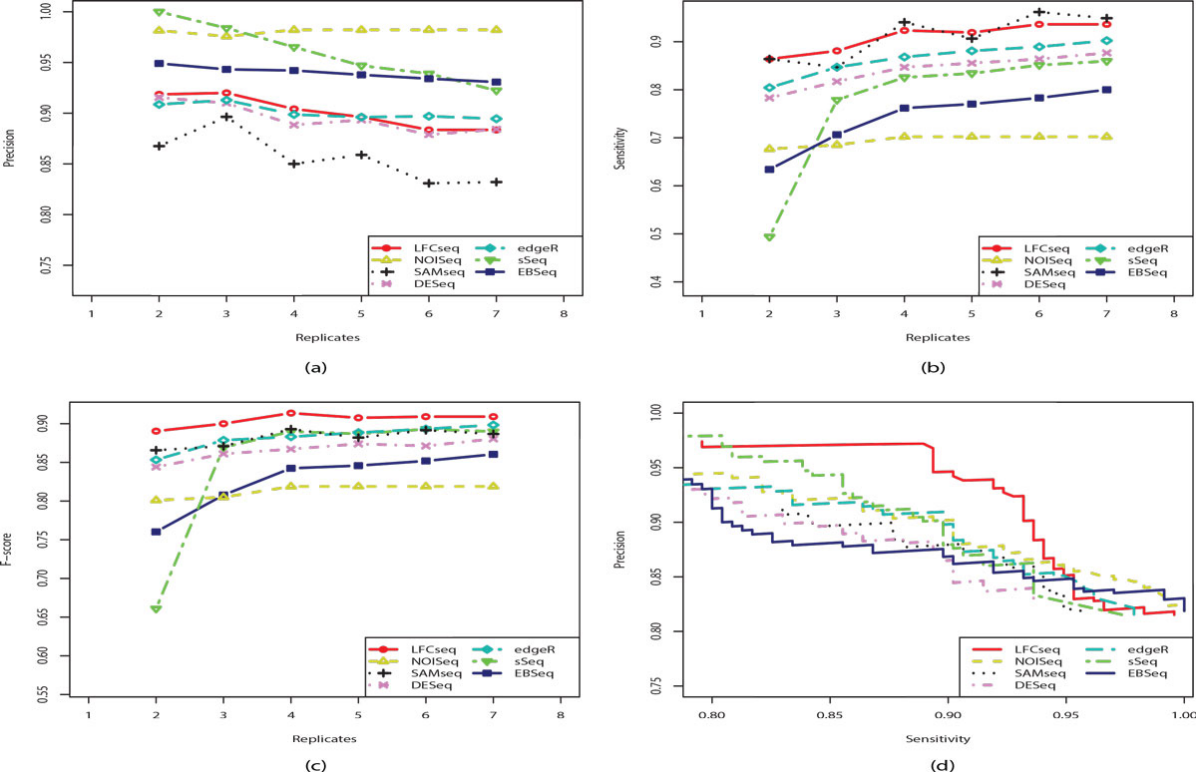
**Comparison of different approaches on MAQC dataset**. (a) Precision curves of LFCseq and six competitors at varying number of replicates. (b) Sensitivity curves. (c) F-score curves. (d) Precision against sensitivity curves when the number of replicates is 7 in each condition.

On MAQC dataset, the curves of precision, sensitivity and F-scores obtained with varying number of replicates, as well as the precision-sensitivity curves obtained with 7 replicates per condition, are shown in Figure 4. Similar to the results in the previous simulation study, LFCseq achieved higher sensitivity and the comparable levels of precision with other methods. As a result, it has the highest F-scores and hence the best overall performance in all the tests. In comparison, NOISeq provides higher precision than LFCseq, but its sensitivity scores are significantly lower than LFCseq's by up to 22%. On the other hand, SAMSeq achieved comparably high sensitivity scores with LFCseq, but its precision scores are always the lowest among all the tested methods. It is interesting to note that SAMSeq behaved differently in the simulation study, where its precision scores are instead higher than LFCseq's in most cases. As the number of replicates increases, NOISeq maintains a relatively stable level of precision while all other approaches lose some precision. This result is in agreement with the observation in [17]. In addition, the precision-sensitivity curves also clearly indicate that LFCseq is a high-performing approach for differential expression analysis of RNA-seq data, as it yields the improved balance between precision and sensitivity.

On Griffith's dataset, the corresponding curves of precision, sensitivity and Fscores are presented in Figures S4-S6 in Additional file 1. Overall, we observed a similar pattern of performance to that observed on MAQC dataset. One noticeable difference is that although LFCseq still achieved the best overall performance in terms of F-score, there are only marginal improvements over the two parametric approaches edgeR and DESeq. Recall that only 11 truly non-DE genes were identified from Griffith's limited RT-PCR data for the validation of prediction results. Such a small true negative dataset is hardly sufficient to fully characterize the performance behavior of a method.

On Sultan's dataset, no gold-standard is available for performance validation. Instead of computing precision and sensitivity scores, we plotted in Figure S7 in Additional file 1 the fold changes of genes against their mean expression levels on the logarithmic scale. In those scatter plots, each red dot represents a gene being called DE while each black dot represents a gene being called non-DE. As we can see, LFCseq called DE genes at both high and low expression ranges. However, NOISeq called few DE genes at low expression ranges, which might suggest that NOISeq is biased against genes with low read counts and that its sensitivity could still be very low as we observed earlier. We also notice that sSeq called a considerably less number of DE genes than other approaches, which indicates that it is very conservative when calling DE genes.

## Conclusions

In this paper we proposed a new nonparametric approach for differential expression analysis of RNA-seq data. It relies on the statistical tests of log fold changes of gene read counts between and within biological conditions. Following the observation that the standard errors of log fold changes vary considerably with gene expression levels, we choose to create a gene-specific null probability distribution for each gene rather than a common null probability distribution for all genes. This is done by considering the gene neighborhood, which is defined as a set of genes at similar expression levels. As a result, the estimated probability of a gene being DE depends only on the read counts of genes from its neighborhood.

Our experimental results demonstrate that the proposed approach LFCseq outperforms its competitors in better ranking the truly DE genes ahead of non-DE genes. It has the best overall performance as it achieved the highest F-scores in almost all our tests (except a few tests on Griffith's dataset). The improvements over other methods are especially remarkable when the number of replicates is small. In such cases, those parametric methods based on negative binomial distribution, such as edgeR, DESeq and sSeq, could not estimate the distributional parameters accurately, while for the nonparametric SAMseq method, its Wilcoxon statistic has a relatively low testing power.

In this study, we applied a pre-specified probability cutoff of 0.1 for our approach LFCseq. This cutoff generally works well, as shown in our experiments on both simulated data and real RNA-seq data. However, it is certainly of interest to develop a data-driven cutoff selection method for a wide applicability of the approach. In addition, it is also interesting to formulate a framework to control the false discovery rate [27] for our approach. We will explore these in future work.

## Competing interests

The authors declare that they have no competing interests.

## Authors' contributions

BQL and XC conceived the idea. BQL and XC contributed to the design of the study. BQL processed the data and conducted simulation and real dataset experiments. BQL, LFZ and XC wrote the manuscript. All authors read and approved the final manuscript.

## Supplementary Material

Additional file 1 **Supplementary text and figures**. This file contains related codes to use existing approaches, information and results for simulated and real datasets. Click here for file

## Declarations

The publication cost for this article was funded by a grant from the Singapore Ministry of Education Academic Research Fund (MOE2012-T2-1-055).

This article has been published as part of *BMC Genomics *Volume 15 Supplement 10, 2014: Proceedings of the 25th International Conference on Genome Informatics (GIW/ISCB-Asia): Genomics. The full contents of the supplement are available online at http://www.biomedcentral.com/bmcgenomics/supplements/15/S10.
