# Supplementary material for: LFCseq: a nonparametric approach for differential expression analysis of RNA-seq data
Source: BMC Genomics. 2014 Dec 12;15(Suppl 10):S7. doi: 10.1186/1471-2164-15-S10-S7 (PMC4304217; doi:10.1186/1471-2164-15-S10-S7)
Supplement: Additional file 1 — Supplementary text and figures. This file contains related codes to use existing approaches, information and results for simulated and real datasets. [file 1471-2164-15-S10-S7-S1.pdf]

LFCseq: a nonparametric approach for differential expression analysis of RNA-seq data - supplementary materials

Bingqing Lin[1,3], Li-Feng Zhang[1], and Xin Chen[2]

[1]School of Biological Sciences,

[2]School of Physical and Mathematical Sciences,

Nanyang Technological University, Singapore

[3]Institute of Statistical Science,

Shenzhen University, Shenzhen, China

1 Use of existing approaches and related codes

All analyses were performed with R-3.0.2 (R Core Team, 2013) on Windows 7. All approaches need two pieces of information, the read count matrix and the conditions. For simplicity, we denote the read count matrix as *seqData* and conditions as *conds*, *conds*=c(*rep*("*A*", *c*1), *rep*("*B*", *c*2)), where $c1 = |A|$ and $c2 = |B|$, respectively.

1.1 NOISeq

The NOISeq (v2.4.0) package can be installed from Bioconductor (Gentleman et al., 2004).

```
> myData <- readData(data = seqData, factors = data.frame(conds = conds))
> mynoiseq <- noiseq(myData, k = 0.5, norm = "tmm", factor = "conds",
    pnr = 0.2, nss = 5, v = 0.02, lc = 1, replicates = "technical")
> pval <- mynoiseq@results[[1]][, "prob"]
```

1.2 SAMseq

The samr (v2.0) package can be installed from R CRAN.

```
> if(is.null(rownames(seqData)))rownames(seqData)=1:nrow(seqData)
> conds12 <- unique(conds)
> y <- conds == conds12[2];  y <- y +1
> mySAMseq <- SAMseq(x = seqData, y = y, resp.type = "Two class unpaired",
    nperms = 100, nresamp = 20,fdr.output = 1,geneid = rownames(seqData),
            genenames = rownames(seqData))
> SAMseq.result.table <- rbind(mySAMseq$siggenes.table$genes.up,
        mySAMseq$siggenes.table$genes.lo)
> myFDR <- rep(1, nrow(seqData))
> names(myFDR) <- rownames(seqData)
> myFDR[match(SAMseq.result.table[,1], names(myFDR))] <-
        as.numeric(SAMseq.result.table[,5])/100
```

1.3 edgeR

The edgeR (v3.4.2) package can be installed from Bioconductor.

```
> myedgeR <- DGEList(counts = seqData, group = conds)
> myedgeR <- calcNormFactors(myedgeR)
> myedgeR <- estimateCommonDisp(myedgeR)
> myedgeR <- estimateTagwiseDisp(myedgeR)
> myedgeRres <- exactTest(myedgeR, dispersion="auto")
> pval <- myedgeRres$table$PValue
```

1.4 DESeq

The DESeq (v1.14.0) package can be installed from Bioconductor.

```
> conds12 <- unique(conds)
> myDESeq <- newCountDataSet(seqData, conds)
> myDESeq <- estimateSizeFactors(myDESeq)
> myDESeq <- estimateDispersions(myDESeq, fitType="local")
> res <- nbinomTest(myDESeq, conds12[[1]], conds12[[2]] )
> pval <- res$pval
> padj <- res$padj
```

1.5 sSeq

The sSeq (v1.0.0) package can be installed from Bioconductor.

```
> conds12 <- unique(conds)
> res <- nbTestSH(seqData, conds, conds12[[1]], conds12[[2]])
> pval <- res$pval
```

1.6 EBSeq

The EBSeq (v1.2.0) package can be installed from Bioconductor.

```
> rownames(seqData) <- paste("gene", 1:nrow(seqData), sep="")
> seqDepth <- MedianNorm(seqData)
> res <- EBTest(Data = as.matrix(seqData), Conditions = as.factor(conds),
        sizeFactors = seqDepth, maxround = 5)
> pp <- GetPPMat(res)
> head(pp)
```

2 Datasets

Simulation 1. In this simulated dataset, there are a total of 20000 genes and their read counts are generated from a negative binomial distribution under each condition A or B,

$$N_{ij} \sim \mathcal{NB}(\mu_{ij}, \sigma^2_{ij})$$

where μ_{ij} and σ^2_{ij} are the mean and variance. We further let $\mu_{ij} = \mathrm{E}\{N_{ij}\} = q_{iA} \cdot d_j$ under condition A and $\mu_{ij} = \mathrm{E}\{N_{ij}\} = q_{iB} \cdot d_j$ under condition B. 30% of the genes are simulated to be differentially expressed, among which 70% are set to be up-regulated. Expected read counts for condition A were randomly sampled from $q_g^A \sim \text{Exponential}(\lambda = 250)$. The dispersion parameter is considered as a constant, $\phi_g = 0.1$. For up-regulated differentially expressed genes, $q_g^B = q_g^A \exp(|\epsilon|)$, for down-regulated differentially expressed genes, $q_g^B = q_g^A \exp(-|\epsilon|)$, for non-differentially expressed genes, $q_g^B = q_g^A$, where $\epsilon \sim N(0, 1)$. The library size factors are generated from the uniform distribution $d_j \sim U(0.5, 1.5)$. We let $|A| = |B| = 2$, 5 and 8 under each condition.

Simulation 2. We generate read counts for $G = 20000$ features using the exactly same way as Simulation 1 except two important parameters (q_g^A, ϕ_g) in negative binomial distribution are randomly sampled from the estimated pairs in experimental Bottomly's dataset. The collection of estimated pairs contains the estimated (q_g, ϕ_g) for a subset of 11123 genes whose average read counts are larger than 1. The subset is the intersection of non-DE subsets selected by SAMseq, edgeR and DESeq.

MAQC dataset. The read counts for the RNA-Seq experiment were downloaded from http://bowtie-bio.sourceforge.net/recount/ (Frazee et al., 2011). qRT-PCR data were downloaded form Gene Expression Omnibus (GEO) (accession GSE5350).

Griffith's dataset. The reads for the RNA-Seq experiment and qPCR data were downloaded from ALEXA-Seq Web site (Griffith et al., 2010). Reads were mapped against the human genome UCSC hg19 (Meyer et al., 2013) using Tophat (Trapnell and andSteven L. Salzberg, 2009). We allowed up to two mismatches and removed reads mapped to multiple locations. The read counts were further computed by two R packages, GenomicFeatures and RSamtools from Bioconductor.

Sultan's Dataset. The read counts for the RNA-Seq experiment were downloaded from http://bowtie-bio.sourceforge.net/recount/ (Frazee et al., 2011).

Bottomly's Dataset. Gene expression of two commonly used inbred mouse strains, C57BL/6J (B6) and DBA/2J (D2), were compared using RNA-Seq (Bottomly et al., 2011). In this dataset, there are 10 replicates for C57BL/6J and 11 replicates for DBA/2J. The read counts for the RNA-Seq experiment were downloaded from
http://bowtie-bio.sourceforge.net/recount/ (Frazee et al., 2011).

3 Results

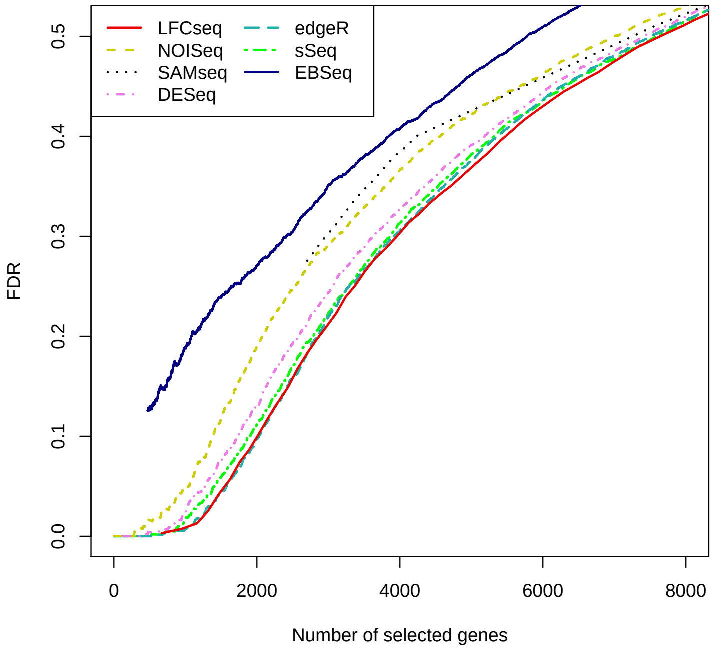

Supplementary Figure S1: False discovery rate curve for Simulation 2, 2 replicates per condition.

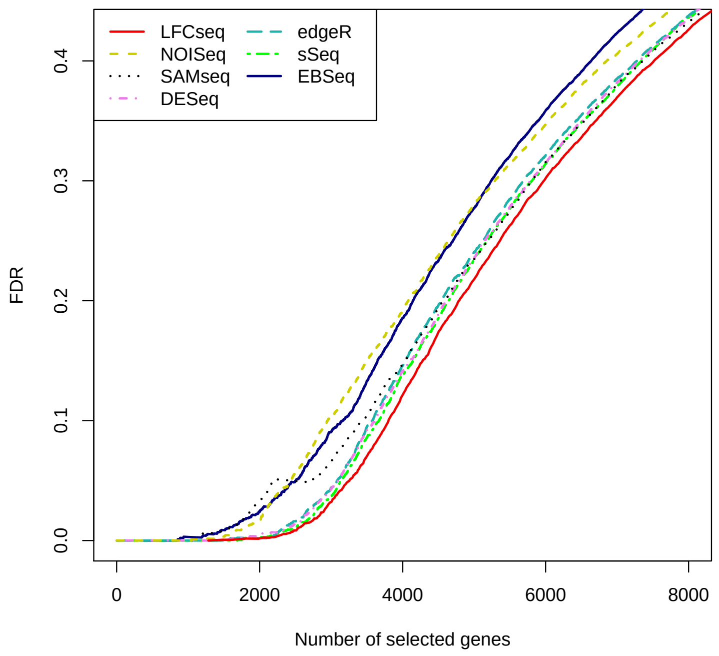

Supplementary Figure S2: False discovery rate curve for Simulation 2, 5 replicates per condition.

Supplementary Table S1: Precision, sensitivity and F-score for Simulation 2. The numbers of replicates per condition are 2, 5 and 8, respectively. The highest precision, sensitivity and F-scores are highlighted in bold.

**Methods**	**PRE**	**SEN**	**FS**	**PRE**	**SEN**	**FS**	**PRE**	**SEN**	**FS**
	$\|A\| = \|B\| = 2$			$\|A\| = \|B\| = 5$			$\|A\| = \|B\| = 8$		
LFCseq	0.86	**0.36**	**0.50**	0.93	**0.53**	**0.67**	0.93	**0.62**	**0.74**
NOISeq	0.97	0.15	0.27	**1.00**	0.15	0.26	**1.00**	0.15	0.26
SAMseq	NA	0.00	NA	0.96	0.31	0.47	0.96	0.54	0.69
DESeq	**0.99**	0.15	0.25	0.99	0.38	0.55	0.98	0.50	0.67
edgeR	0.97	0.25	0.39	0.95	0.47	0.63	0.94	0.56	0.71
sSeq	NA	0.00	NA	0.97	0.43	0.60	0.95	0.56	0.70
EBSeq	0.73	0.27	0.39	0.95	0.36	0.52	0.98	0.44	0.60

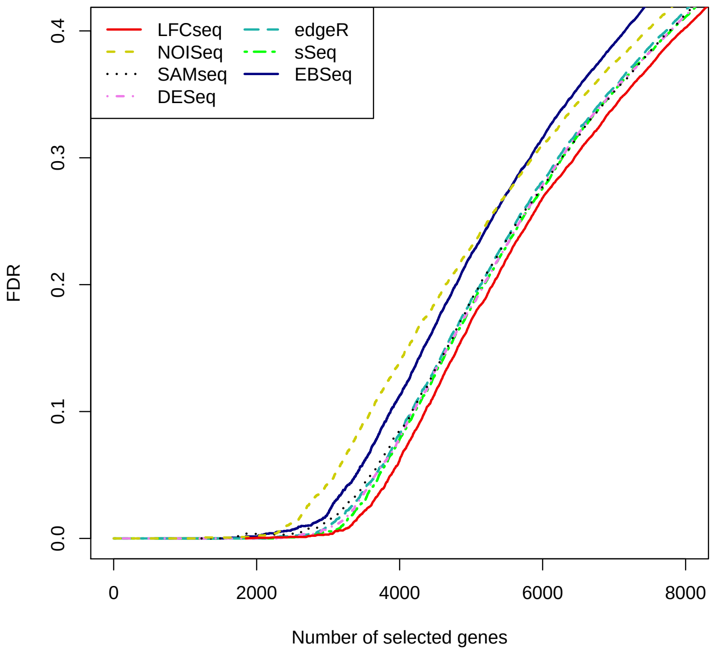

Supplementary Figure S3: False discovery rate curve for Simulation 2, 8 replicates per condition.

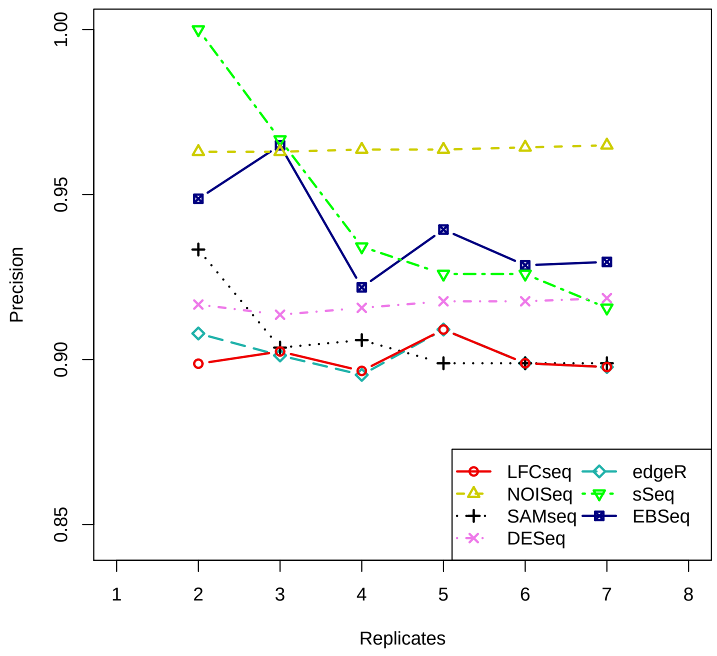

Supplementary Figure S4: Precision curves of LFCseq and six competitors at varying number of replicates on Griffith's dataset.

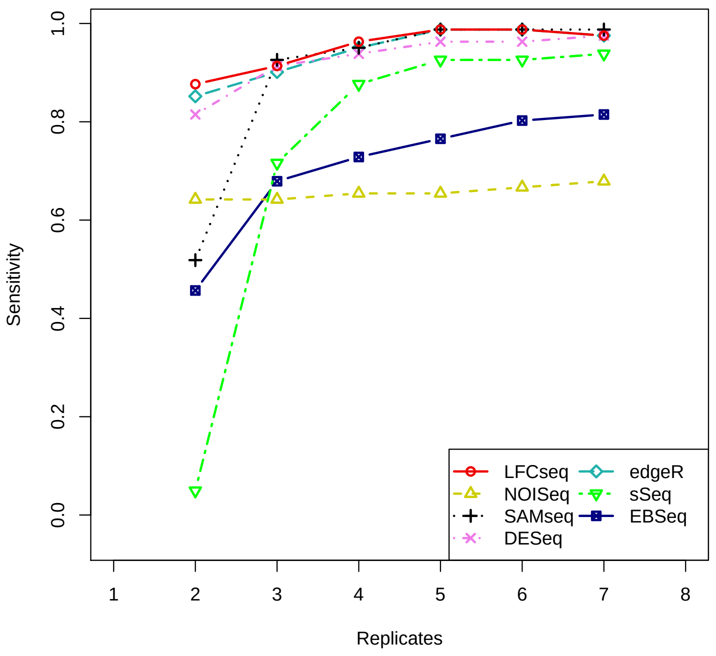

Supplementary Figure S5: Sensitivity curves of LFCseq and six competitors at varying number of replicates on Griffith's dataset.

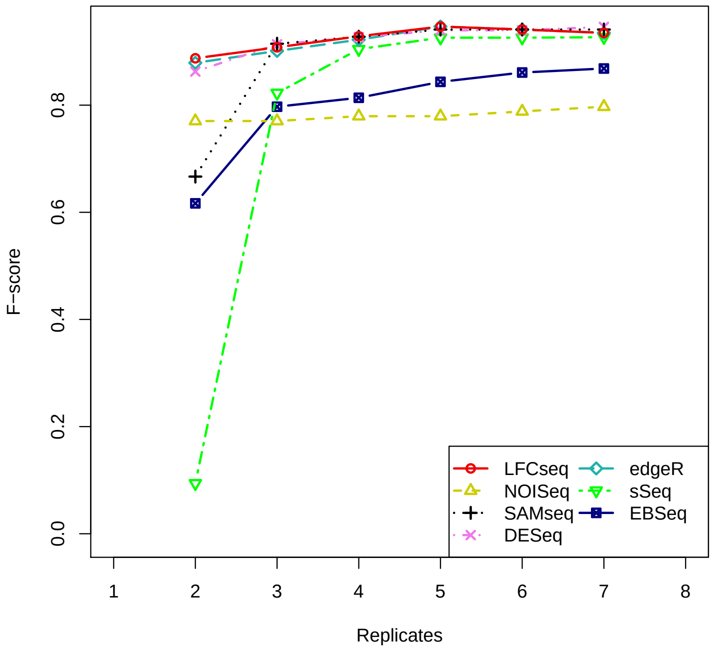

Supplementary Figure S6: F-score curves of LFCseq and six competitors at varying number of replicates on Griffith's dataset.

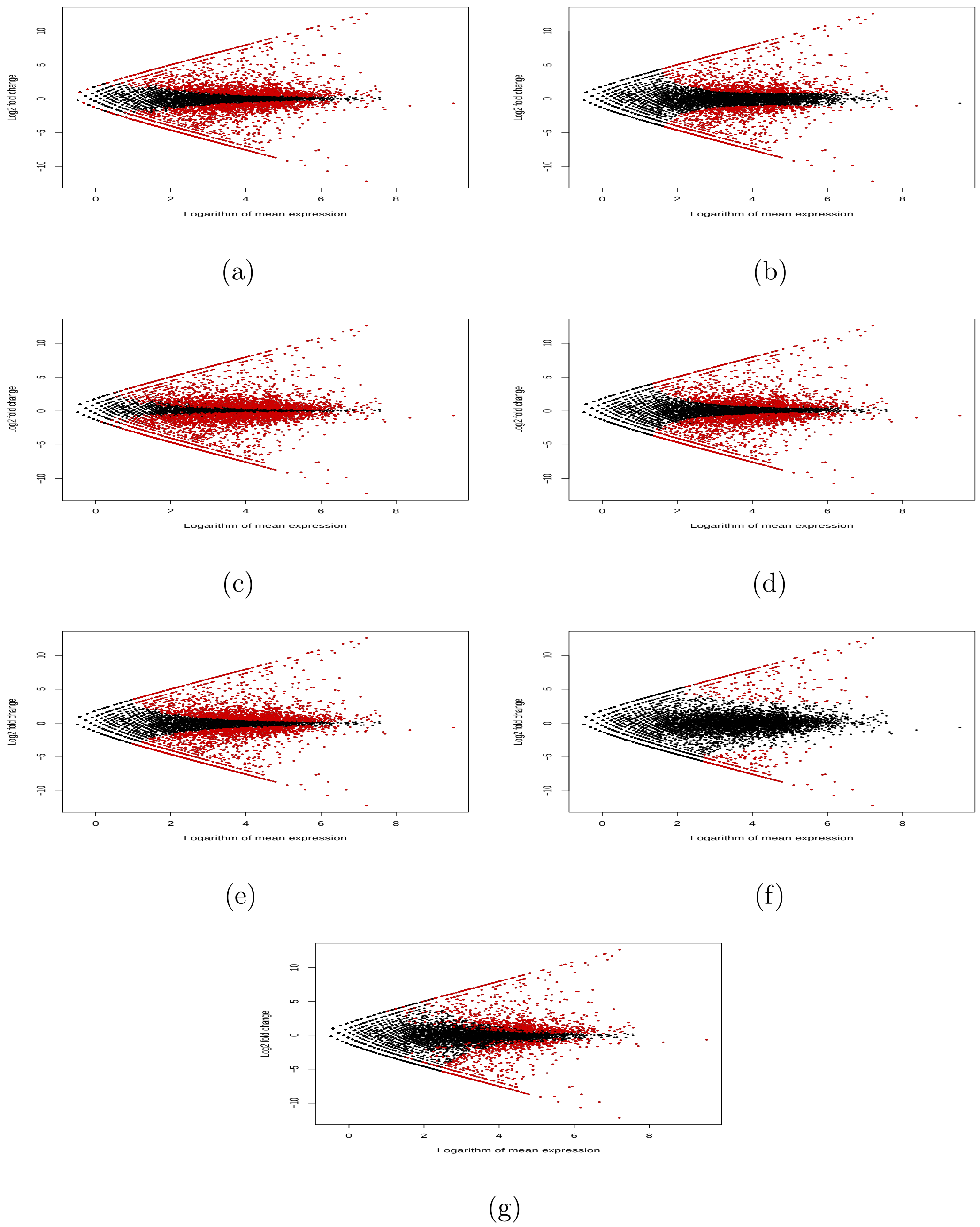

Supplementary Figure S7: Differential expression between cell lines Ramos B and HEK 293T: log fold change versus logarithm of mean expression. Each red dot represents a gene being called DE while each black dot represents a gene being called non-DE. (a) LFCseq. (b) NOISeq. (c) SAMseq. (d) DESeq. (e) edgeR. (f) sSeq. (g) EBSeq.

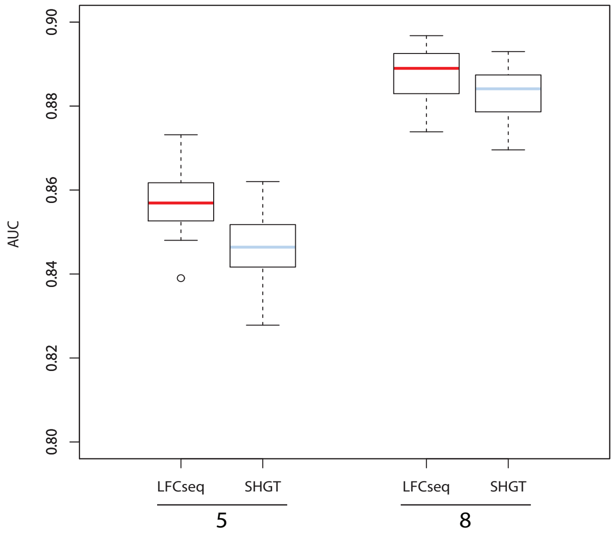

Supplementary Figure S8: Areas under the ROC curves for Simulation 1 to compare LFCseq with a simple hypergeometric test (SHGT). The numbers of replicates per condition are 5 and 8, respectively. In the simple hypergeometric test, null distribution for gene i is built on the randomly permuted samples between conditions A and B of gene i. When the total number of replicates of the dataset is less than 10, we built the null distribution using all possible permutations. When the total number of replicates of the dataset is larger than 10, we compute it for 500 random permutations to reduce the computational cost.

Supplementary Table S2: Precision, sensitivity and F-score for Simulation 1 to compare LFCseq with simple hyper geometric test (SHGT). The numbers of replicates per condition are 5 and 8, respectively. Both approaches used a probability cutoff, 0.1.

Methods	PRE	SEN	FS	PRE	SEN	FS
	$\|A\| = \|B\| = 5$			$\|A\| = \|B\| = 8$		
LFCseq	0.934	0.590	0.723	0.935	0.683	0.789
SHGT	0.752	0.678	0.713	0.758	0.762	0.760

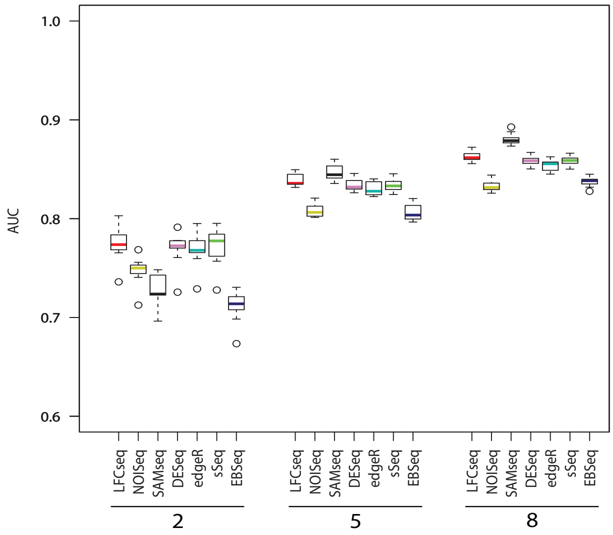

Supplementary Figure S9: Areas under the ROC curves for Simulation 1 with outliers. We let 0.5% of observed read counts become outliers. That is, we multiplied randomly selected 0.5% observed read counts with randomly generated numbers between 5 and 10. As expected, all methods except SAMseq performs worse for Simulation 1 with outliers than Simulation 1 without outliers. LFCseq has comparable or slightly higher AUC values compared with other approaches except SAMseq for all situations. And, LFCseq achieves higher AUC values than SAMseq when the number of replicates for each condition is 2.

Supplementary Table S3: Average of computational time over 20 repetitions for Simulation 1.

Methods	Computational Time (s)		
	$\|A\| = \|B\| = 2$	$\|A\| = \|B\| = 5$	$\|A\| = \|B\| = 8$
LFCseq	156	213	184
NOISeq	76.8	457	912
SAMseq	9.10	42.7	33.5
DESeq	40.1	100	118
edgeR	11.72	16.4	15.5
sSeq	53.68	122	134
EBSeq	80.4	245	292

Supplementary Table S4: AUC values for MAQC dataset. The numbers of replicates per condition are from 2 to 7.

	LFCseq	NOISeq	SAMseq	DESeq	edgeR	sSeq	EBSeq
$\lvert A \rvert = \lvert B \rvert = 2$	0.872	0.856	0.756	0.816	0.828	0.864	0.831
$\lvert A \rvert = \lvert B \rvert = 3$	0.876	0.866	0.762	0.828	0.842	0.874	0.826
$\lvert A \rvert = \lvert B \rvert = 4$	0.881	0.872	0.789	0.831	0.850	0.878	0.831
$\lvert A \rvert = \lvert B \rvert = 5$	0.877	0.871	0.782	0.831	0.848	0.881	0.823
$\lvert A \rvert = \lvert B \rvert = 6$	0.862	0.873	0.765	0.829	0.849	0.882	0.820
$\lvert A \rvert = \lvert B \rvert = 7$	0.884	0.876	0.748	0.829	0.856	0.882	0.820

References

Bottomly, D., Walter, N. A. R., and Huner, J. E. (2011). Evaluating gene expression in c57bl/6j and dba/2j mouse striatum using rna-seq and microarrays. *Plos One*, 6:e17820.

Frazee, A. C., Langmead, B., and Leek, J. T. (2011). Recount: A multi-experiment resource of analysis-ready rna-seq gene count datasets. *BMC Bioinformatics*, 12:449.

Gentleman, R. C., Carey, V. J., Bates, D. M., Bolstad, B., Dettling, M., Dudoit, S., Ellis, B., Gautier, L., Ge, Y., Gentry, J., Hornik, K., Hothorn, T., Huber, W., Iacus, S., Irizarry, R., Leisch, F., Li, C., Maechler, M., Rossini, A. J., Sawitzki, G., Smith, C., Smyth, G., Tierney, L., Yang, J. Y., and Zhang, J. (2004). Bioconductor: open software development for computational biology and bioinformatics. *Genome Biology*, 5:R80.

Griffith, M., Griffith, O., and Mwenifumbo, J. (2010). Alternative expression analysis by rna sequencing. *Nature Methods*, 7:843–847.

Meyer, L. R., Zweig, A. S., Hinrichs, A. S., Karolchik, D., Kuhnv, R. M., Wong, M., Sloan, C. A., Rosenbloom, K. R., Roe, G., Rhead, B., Raney, B. J., Pohl, A., Malladi, V. S., Li, C. H., Lee, B. T., Learned, K., Kirkup, V., Hsu, F., Heitner, S., Harte, R. A., Haeussler, M., Guruvadoo, L., Goldman, M., Giardine, B. M., Fujita, P. A., Dreszer, T. R., Diekhans, M., Cline, M. S., Clawson, H., Barber, G. P., Haussler, D., and and, W. J. K. (2013). The ucsc genome browser database: extensions and updates 2013. *Nucleic Acids Research*, 41:D64–D69.

R Core Team (2013). *R: A language and environment for statistical computing. R Foundation for Statistical Computing.* Vienna, Austria. URL http://www.R-project.org/.

Trapnell, C. and andSteven L. Salzberg, L. P. (2009). Tophat: discovering splice junctions with rna-seq. *Bioinformatics*, 25:1105–1111.
